# LncRNA Gas5 acts as a ceRNA to regulate PTEN expression by sponging miR-222-3p in papillary thyroid carcinoma

**DOI:** 10.18632/oncotarget.23336

**Published:** 2017-12-16

**Authors:** Xiao-Fang Zhang, Yan Ye, Shu-Jun Zhao

**Affiliations:** ^1^ Key Laboratory of Hormones and Development (Ministry of Health), Tianjin Key Laboratory of Metabolic Diseases, Tianjin Metabolic Diseases Hospital and Tianjin Institute of Endocrinology, Tianjin Medical University, 300070 Tianjin, China

**Keywords:** Gas5, competing endogenous RNA, PTEN, papillary thyroid carcinoma, proliferation

## Abstract

Accumulating evidence demonstrates that the long non-coding RNA Growth Arrest-Specific 5 (Gas5) has practical significance in cancer progression and metastasis. However, its role and function in papillary thyroid carcinoma (PTC) remains unknown. In this study, we aimed to explore the potential involvement of Gas5 in papillary thyroid carcinogenesis and to highlight the emerging roles of ceRNAs in the biological regulation of PTC cells. The results suggested that Gas5 was markedly downregulated in both PTC tissues and PTC cell lines. Over-expression of Gas5 remarkably suppressed PTC cells proliferation *in vitro* and inhibited the growth of tumor cells *in vivo* likewise. Furthermore, Gas5 was identified as a target of miR-222-3p which was aberrantly high in PTC cells. Enhanced expression of miR-222-3p promoted the proliferation of PTC cells while knocking down miR-222-3p could inhibit it. The advanced effects of miR-222-3p on the proliferation of PTC cells could be partly reversed by the upregulation of Gas5 expression. Furthermore, we validated that Gas5 increased the protein level of the PTEN, one of miR-222-3p’s targets, which further activated PTEN/AKT pathway. Taken together, our study identified a tumor suppressive role of Gas5 in PTC cells acting as a ceRNA, effectively becoming a sink for miR-222-3p, modulating the expression of PTEN, which lead to PTEN/AKT pathway activation and proliferation suppression. This finding may offer a new potential therapeutic strategy for PTC.

## INTRODUCTION

Thyroid cancer, with an increasing incidence in recent years, is the most common type of endocrine malignancies [1–3]. It is classified into four histologic types: papillary, follicular, medullary, and anaplastic thyroid carcinoma. Papillary thyroid carcinoma (PTC) is the main form of thyroid cancer, accounting for greater than 80% of all thyroid malignancies mainly in young women and children [4]. Although PTC has a favorable prognosis for patients in early stages, in which the overall five-year survival rate is 97%, patients with advanced thyroid cancer only have a five-year survival rate of about 59% [5]. Therefore, it is essential to identify more effective therapeutic strategies.

Recently, people have paid more attention on long non-coding RNAs (lncRNAs). As a new modulator, lncRNAs have been shown to regulate tumor progression [6]. Numerous data have showed that lncRNAs (defined as >200 nt) are dysregulated in cancer biology [7]. In some pathological processes, such as cancer, the expression levels of lncRNAs are increased (e.g., HOTAIR) or decreased (e.g., Gas5) [8–10]. Once a report demonstrated that dysregulated expressions of key lncRNAs in ceRNA (competing endogenous RNA) network focused on the miRNA-mediated lncRNA/mRNA crosstalk and broke bistable condition [11]. Gas5 (Growth Arrest-Specific 5) has been first observed by a study that demonstrated a tumor suppression role in 1988. Since then, Gas5 became a star gene among researchers [12, 13]. Accumulating data showed that Gas5 could be implicated in the tumorigenesis and progression of many cancers and may be a useful diagnostic and prognostic cancer biomarker [14]. However, to date, the role of Gas5 in PTC remains largely unexplored. Therefore, the present study aims to explore the potential role of Gas5 in papillary thyroid carcinogenesis and to highlight the emerging roles of ceRNAs in the biological regulation of PTC cells.

PTEN (phosphatase and tension homologue) has been identified as a tumor suppressor gene to be involved in the multi-step biological processes, such as focal adhesion, migration, and proliferation of cancer cells [15, 16]. MiRNAs were reported deregulated and promoted cancer development by down-regulating PTEN expression in some cancers [17]. Among them, the expression of miR-222-3p and its target gene PTEN, as well as their relationship has been established in PTC. Low level of PTEN activity induces phosphorylation of AKT, promoting cell proliferation and migration [18–20].

In this study, we verified the tumor suppressive role of Gas5 as a ceRNA in PTC to modulate PTEN level and identified miR-222-3p as the specific miRNA decoyed by Gas5. Furthermore, we highlighted that Gas5 activated PTEN/AKT pathway repressing the proliferation of PTC cells.

## RESULTS

### Gas5 was down-regulated in papillary thyroid carcinoma (PTC) tissue specimens and PTC cell lines

**Figure 1:**
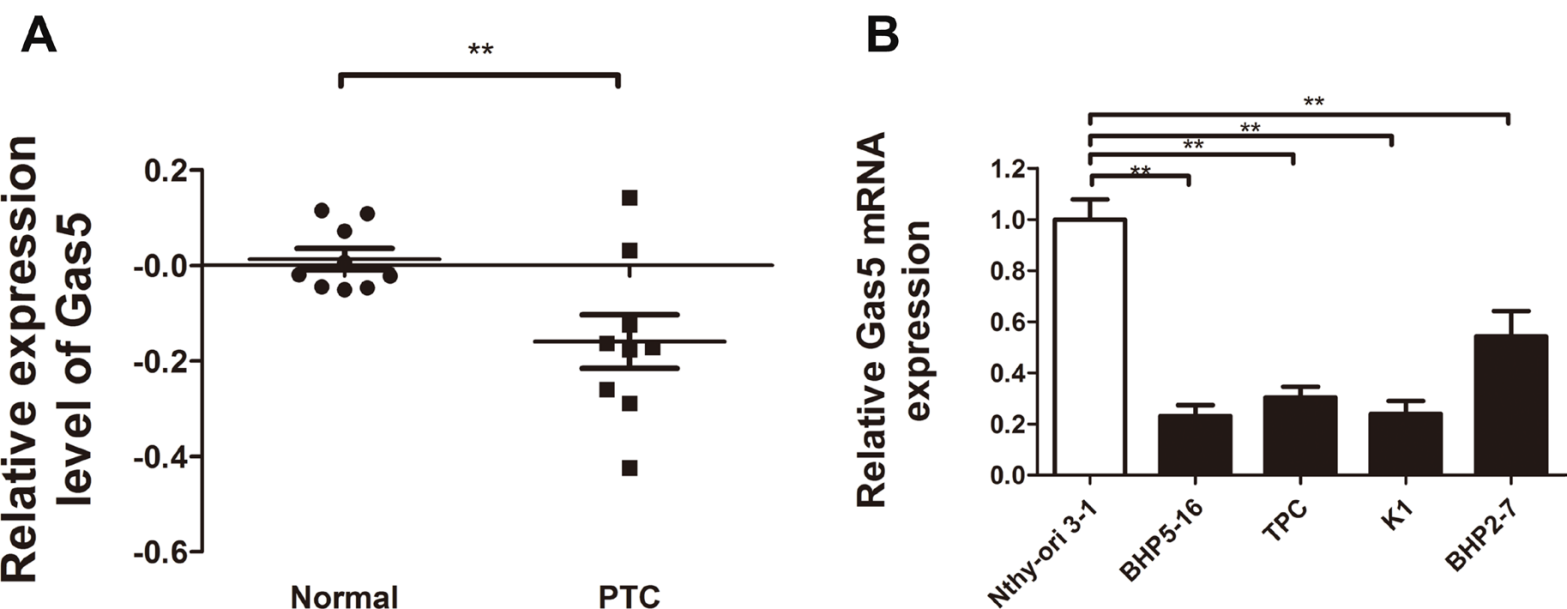
Relative Gas5 expression in papillary thyroid carcinoma (PTC) tissue specimens and PTC cell lines (**A**) Gas5 expression was significantly decreased in human PTC tissue specimens as compared with healthy thyroid tissues (*P* = 0.008). Nine paired thyroid gland papillary carcinoma and normal thyroid gland samples were analyzed in He datasets (Proc Natl Acad Sci U S A 2005). (**B**) Quantitative RT-PCR analysis of Gas5 in PTC cell lines (BHP5-16, TPC, K1, BHP2-7) compared with a normal human thyroid cell line Nthy-ori3-1. ^**^P < 0.01, compared with control.

Numerous evidence links Gas5 dysregulation to human cancers. To verify whether the expression of Gas5 is reduced in PTC clinical tissue specimens, we conducted a review of previous microarray data for the expression of Gas5 on mRNA level in human PTC. Results of researches in Oncomine database (www.oncomine.com) (Rhodes *et al*., 2004) (Figure 1A) revealed that expression of Gas5 was markedly decreased in human PTC as compared with healthy thyroid tissues as shown in He datasets (Proc Natl Acad Sci U S A 2005) (*p* = 0.008).

Furthermore, to confirm the reduced expression of Gas5 in PTC cell lines, we performed qRT-PCR analysis to detect the Gas5 mRNA expression in 4 PTC cell lines (BHP5-16, TPC, K1, BHP2-7) and a normal human thyroid cell line Nthy-ori 3-1. As illustrated in Figure 1B, Gas5 mRNA expression level was significantly lower in four PTC cell lines as compared with Nthy-ori 3-1 (*P* < 0.01, respectively).

These results provide a strong support for the idea that Gas5, which is commonly decreased in many cancers and associated with clinic-pathological characteristics, is frequently reduced in PTC.

### Gas5 represses proliferation of PTC cells *in vitro* and *in vivo*

The remarkable decrease of Gas5 expression in PTC tissue samples and PTC cell lines stimulated us to evaluate the possible mechanism of Gas5 in pathological process of PTC.

**Figure 2:**
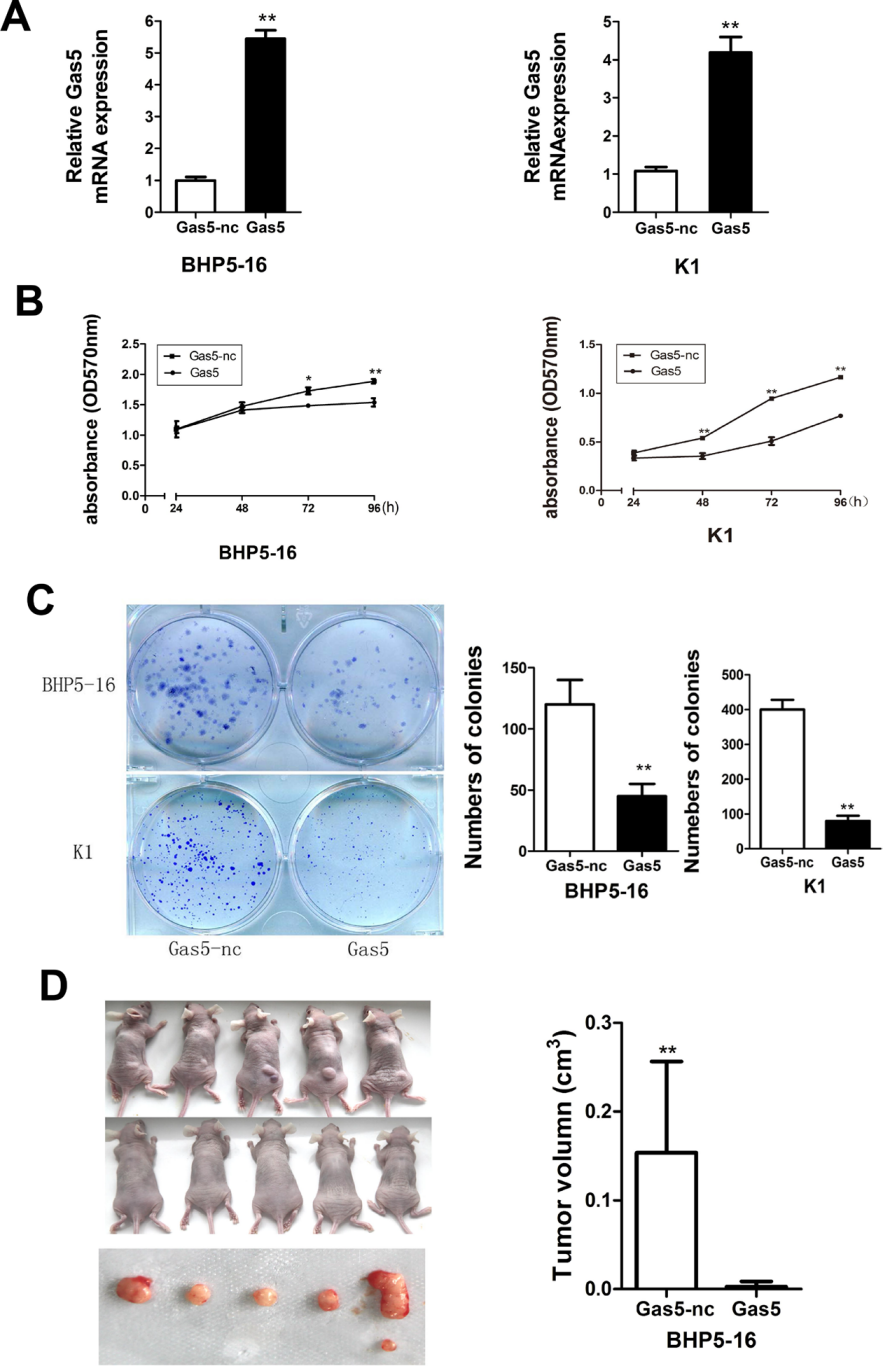
Gas5 represses proliferation of PTC cells *in vitro* and *in vivo* (**A**) QRT-PCR analysis of Gas5 expression level following treatment BHP5-16 and K1 cells with pcDNA3.1/Gas5 vector. (**B**) The proliferation of pcDNA3.1/Gas5 vector–transfected BHP5-16 and K1 cells were detected by MTT assay. (**C**) Colony formation assay was performed to determine the proliferation of pcDNA3.1/Gas5 vector–transfected BHP5-16 and K1 cells. Colonies were counted and captured. Data represent the mean ± SD from three independent experiments. (**D**) Tumor formation assay was conducted in female BALB/c nude mice by subcutaneous injection of BHP5-16 cells (5 × 10^5^) transfected with pcDNA3.1/Gas5 or pcDNA3.1 into the back of the mice. After 6 weeks, tumor tissues were harvested and tumor volumes were measured. ^**^P < 0.01 compared with control.

To enhance Gas5 expression in PTC cells, a pcDNA3.1/Gas5 vector was constructed and transfected into BHP5-16 and K1 respectively. The cells transfected with pcDNA3.1 plasmid were acted as a control. QRT-PCR analysis of Gas5 mRNA levels was performed at 48h post-transfection and revealed that Gas5 expression was increased 5.4-fold in BHP5-16 cells and 4.2-fold in K1 cells, as compared with control group respectively (Figure 2A). Then MTT and colony formation assays were performed to investigate the function of Gas5 on cell proliferation *in vitro*. In MTT assays, results showed that the cell proliferation of BHP5-16 and K1 were significantly decreased in pcDNA3.1/Gas5 transfected cells compared with the control respectively (Figure 2B). Likewise, the results of colony formation assays showed that clonogenic survival was dramatically decreased following forced expression of Gas5 in both BHP5-16 and K1 cells (Figure 2C). To explore whether the level of Gas5 expression affects tumorigenesis, BHP5-16 cells transfected with pcDNA3.1/Gas5 or pcDNA3.1 were used in xenograft model of the nude mice. Up to 6 weeks after injection, tumors were found in all of mice in pcDNA3.1 group while only one mouse bore a tumor in pcDNA3.1/Gas5 group. The tumor volume of pcDNA3.1/Gas5 group is dramatically decreased compared with pcDNA3.1 group (Figure 2D).

In summary, these results show that forced expression of Gas5 can repress proliferation capacity of PTC cells not only *in vitro* but also *in vivo*. Nevertheless, the detailed mechanism by which Gas5 functions need to be further investigated.

### Gas5 is a target of miR-222-3p

**Figure 3:**
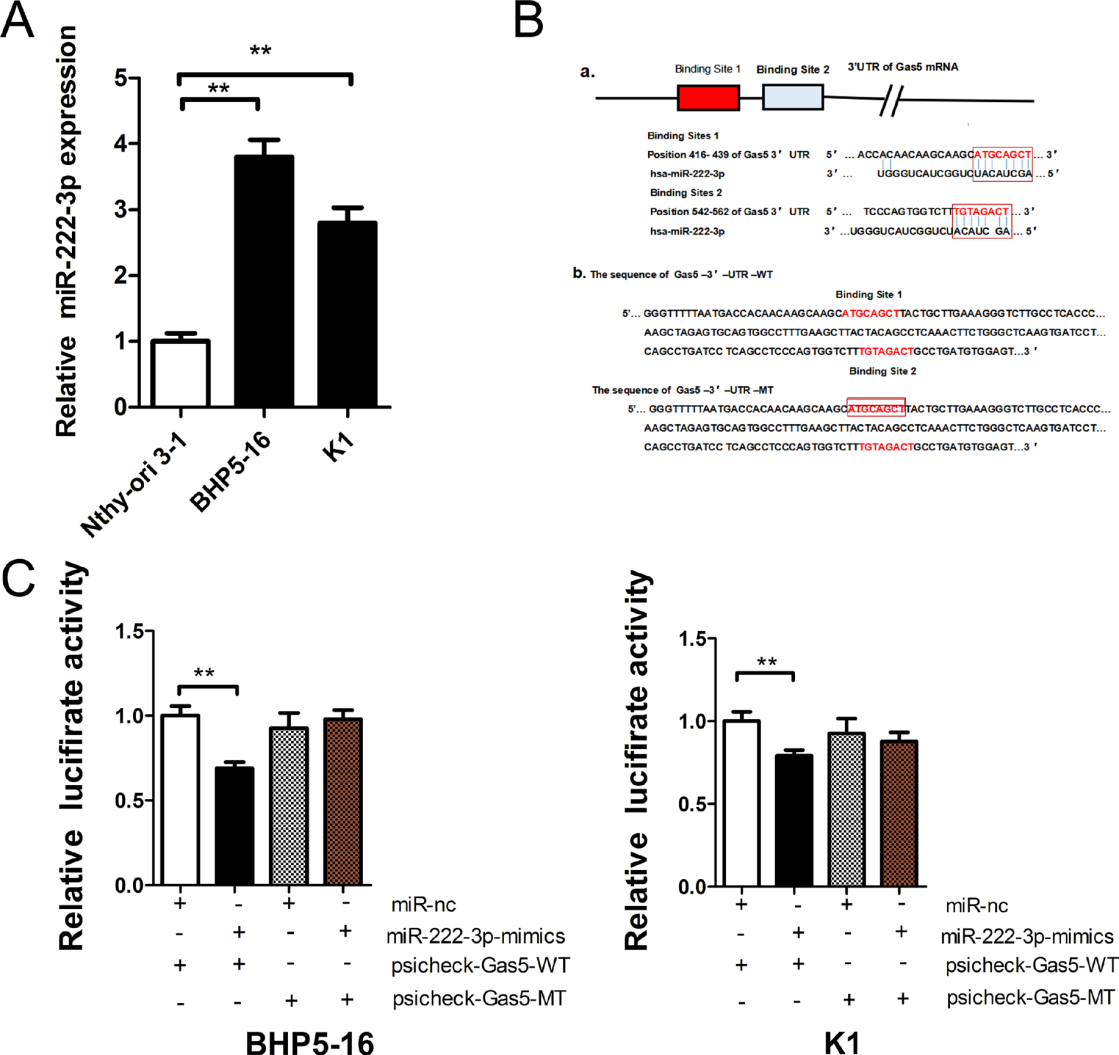
Gas5 is a target of miR-222-3p (**A**) The expression level of endogeneous miR-222-3p in BHP5-16 and K1 cell. (**B**) a: Two miR-222-3p binding sites in Gas5 3′-UTR predicted with software. b: Wild (Gas5-3′-UTR-WT) and mutant (Gas5-3′-UTR-MT) Gas5 3′-UTRs carried in recombinant luciferase mRNAs transcribed by RLuc-Gas5-3′-UTR-WT and RLuc-Gas5-3′- UTR-MT. The binding site 1 was deleted from Gas5-3′-UTR-MT. (**C**) The luciferase reporter plasmid containing wild or mutant-type Gas5 3′-UTR was co-transfected into BHP5-16 and K1 cells with miR-222-3p mimics in parallel with miR-nc. Histogram indicate values of luciferase measured 48 h after transfection. Data represent the mean ± SD from three independent experiments. ^**^P < 0.01 compared with control.

Previous reports demonstrated that lncRNA might act as a ceRNA to miRNA [21]. But accurate regulatory mechanisms of Gas5 remains unclear. Bioinformatics analysis (Starbase 2.0, RNA22) of miRNA recognition sequences on Gas5 revealed the presence of more than 30 miRNAs binding sites. Among them, miR-222-3p stood out through detailed survey, which was considered to facilitate tumor accelleration effect in PTC [22, 23]. Meanwhile, we detected the expression level of endogeneous miR-222-3p in BHP5-16 and K1 cells by qRT-PCR and the results demonstrated that it was drastically increased in both cell lines compared with Nthy-ori 3-1 cell (Figure 3A). Starbase 2.0 and RNA22 predictions revealed that the 3′-UTR of Gas5 mRNA contained two miR-222-3p binding sites (Figure 3B(a)). And then, to experimentally confirm that Gas5 was a target of miR-222-3p, we constructed two recombinant luciferase reporter vectors of Gas5 3′-UTR, i.e., RLuc-Gas5-3′-UTR-WT and RLuc-Gas5-3′-UTR-MT. The recombinant luciferase mRNA transcribed by RLuc-Gas5-3′- UTR-WT carried all miR-222-3p binding sites (Gas5-3′-UTR-WT) predicted in Gas5 3′-UTR while the one transcribed by RLuc-Gas5-3′-UTR-MT lacked the predicted binding site 1 (Gas5-3′-UTR-MT) (Figure 3B(b)). Considering the low value of binding energy predicted by software, we didn’t mutate the second binding site. Co-transfection of RLuc-Gas5-3′-UTR-WT and miR-222-3p mimics was conducted in both BHP5-16 and K1 cells. Co-transfection with RLuc-Gas5-3′-UTR-WT and miR-nc served as a control. The results demonstrated that luciferase activity was reduced by nearly 20% in miR-222-3p co-transfection group compared with the control. To further confirm that the decrease of luciferase activity in the RLuc-Gas5-3′-UTR-WT vector co-transfected cells was due to direct interaction between the miR-222-3p and its putative binding site, we transfected BHP5-16 and K1 with RLuc-Gas5-3′-UTR-MT (lacking the predicted binding site 1) (Gas5-3′-UTR-MT) and miR-222-3p mimics to monitor the luciferase activity. The results revealed that the suppression of luciferase activity was completely abolished in RLuc-Gas5-3′-UTR-MT and miR-222-3p mimics co-transfection cells (Figure 3C).

Taken together, these data suggest that miR-222-3p can directly bind to Gas5 through miRNA recognition sites. As a target of miR-222-3p, Gas5 may act as a ceRNA to miR-222-3p.

### MiR-222-3p promotes proliferation of PTC cells *in vitro*

**Figure 4:**
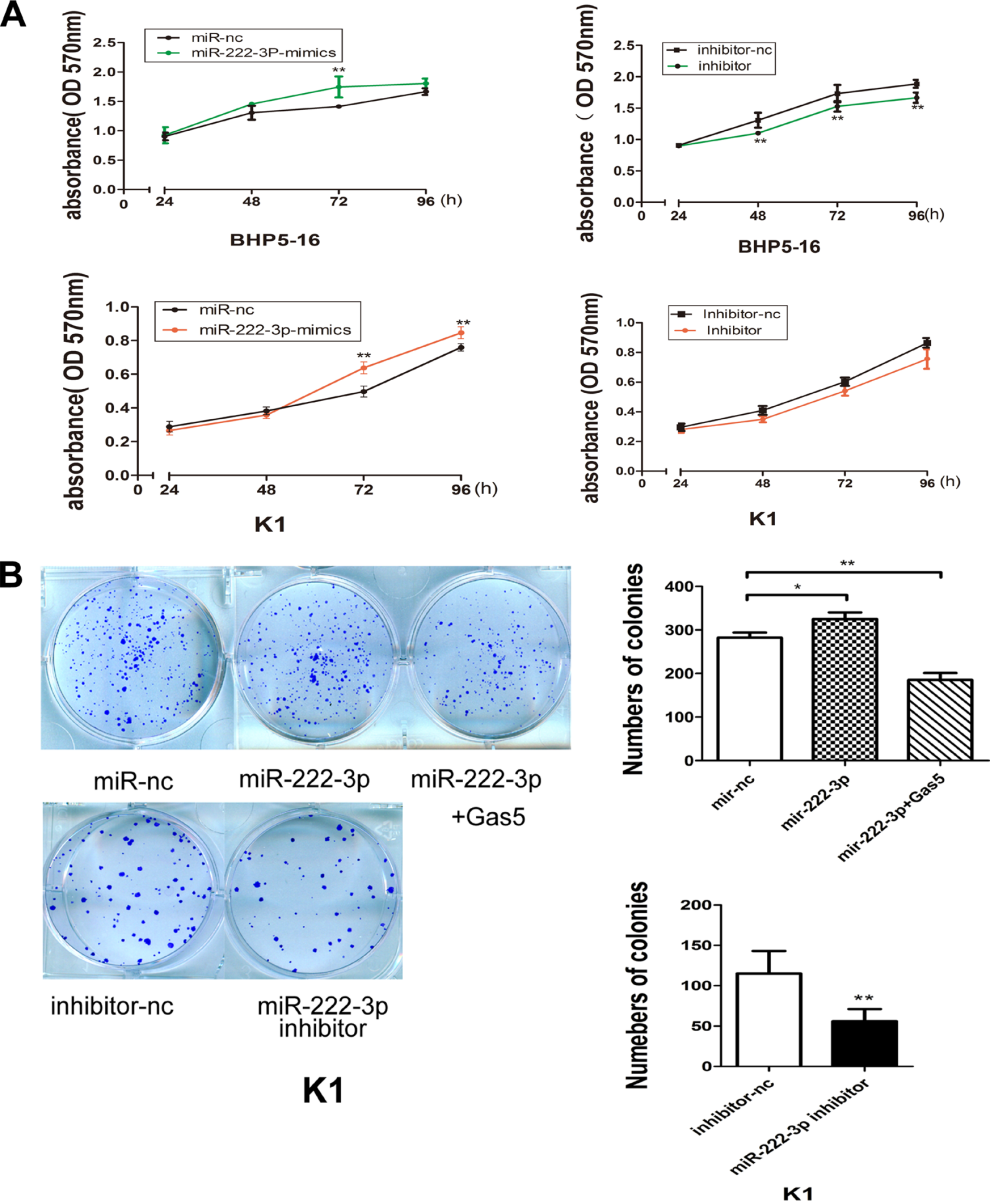
MiR-222-3p promotes proliferation of PTC cells *in vitro* (**A**) Cell proliferation assay of BHP5-16 and K1 transfected with miR-222-3p mimics/inhibitor, the OD values were detected each day at the same time point by MTT assay. (**B**) Colony formation assays were performed to determine the proliferation of K1 cells transfected with miR-222-3p mimics, inhibitor or miR-222-3p mimics plus pcDNA3.1/Gas5. Data are expressed as mean ± SD from three independent experiments. ^*^P < 0.05, ^**^P < 0.01.

By the analysis of the genome-wide miRNAs expression profile in human papillary thyroid carcinoma (PTC), researchers found an over-expression of miR-146b-5p, miR-2223-p and some other miRNAs in PTCs that clearly differentiates PTCs from normal thyroid tissues [22, 23]. To investigate the role of miR-222-3p on PTC carcinogenesis, miR-222-3p mimics or inhibitor was transfected into both BHP5-16 and K1 cells and the proliferation curves were performed using MTT assays. Our results showed that over-expression of miR-222-3p markedly promoted the cell growth in both BHP5-16 and K1 cells when compared with cells transfected with miR-nc. Conversely, the cells transfected with miR-222-3p inhibitor grew at a dramatically lower rate as compared with controls (Figure 4A). Also, colony formation capacity was detected in miR-222-3p mimics, inhibitor or miR-222-3p mimics plus pcDNA3.1/Gas5 treated K1 cells. We observed an enhanced colonigenic capacity in miR-222-3p over-expression cells as compared with controls, which could be reversed by Gas5 co-transfection. By contrast, a dramatically reduction of colony forming capacity has been demonstrated in miR-222-3p inhibitor transfected cells as compared with controls (Figure 4B).

Collectively, these data indicate that miR-222-3p can accelerate PTC cells proliferation, which inversely correlates with the effects of Gas5 in PTC cells.

### Gas5 controls the miR-222-3p’s target, PTEN

**Figure 5:**
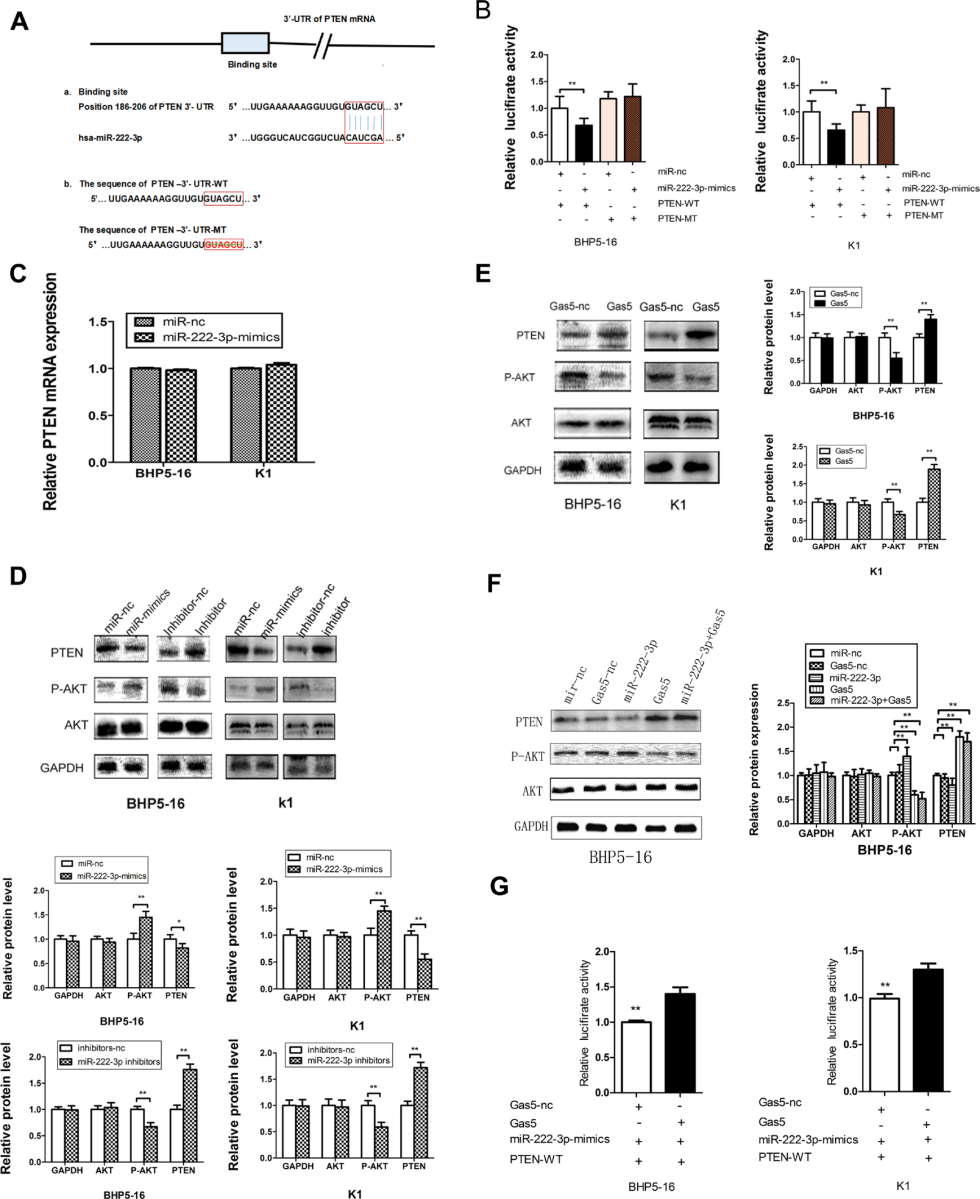
Gas5 controls the miR-222-3p’s target, PTEN (**A**) a: The binding site between miR-222-3p and the 3′-UTR of PTEN mRNA predicted by bioinformatics. b: Wild (PTEN-3′-UTR-WT) and mutant (PTEN-3′-UTR-MT) PTEN 3′-UTRs carried in recombinant luciferase mRNAs transcribed by RLuc-PTEN-3′-UTR-WT and RLuc-PTEN-3′- UTR-MT. The binding site was deleted from PTEN-3′-UTR-MT. (**B**) The luciferase reporter plasmid containing wild or mutant-type PTEN-3′-UTR was co-transfected into BHP5-16 and K1 cells with miR-222 -3p mimics in parallel with miR-nc. (**C**) The mRNA level of PTEN in BHP5-16 and K1 cells transfected with miR-222-3p-mimics or miR-nc. (**D**) PTEN protein levels were detected by western blot in BHP5-16 and K1 cells after transfection with miR-222-3p mimics or miR-222-3p inhibitor. Meanwhile, the total and phosphorylated AKT were examined by western blot. GAPDH was used as an internal control. Similar results were obtained in three independent experiments. (**E**) Western blot analysis of PTEN, total AKT and phosphorylated AKT protein level following treatment of BHP5-16 cells and K1 with pcDNA3.1/Gas5 vector. GAPDH was used as control. (**F**) Western blot analysis of PTEN, total AKT and phosphorylated AKT protein level following treatment of BHP5-16 cells with pcDNA3.1/Gas5 vector and miR-222-3p mimics. (**G**) Rluc-PTEN 3′-UTR-WT and miR-222-3p mimics were co-transfected into BHP5-16 and K1 cells with plasmids expressing pcDNA3.1/Gas5 vector or with a control vector to verify the ceRNA activity of Gas5. Histogram indicate values of luciferase measured 48h after transfection. Data represent the mean ± SD from three independent experiments. ^**^P < 0.01.

Among the many predicted targets of miR-222-3p by bioinformatics software (TargetScan, MiRanda), we focused on PTEN. PTEN is known to act as a tumor suppressor protein, which inhibits the PI3K/AKT pathway, and regulating cell processes including growth, proliferation and polarization [24]. The predicted binding site of miR-222-3p on the 3′-UTR of PTEN mRNA is shown in Figure 5A(a). And then, to experimentally confirm that PTEN was a target of miR-222-3p, we constructed two recombinant luciferase reporter vectors of PTEN 3′-UTR, i.e., RLuc-PTEN-3′-UTR-WT and RLuc-PTEN-3′-UTR-MT. The recombinant luciferase mRNA transcribed by RLuc-PTEN-3′-UTR-WT carried all miR-222-3p binding sites (PTEN-3′-UTR-WT) predicted in PTEN 3′-UTR while the one transcribed by RLuc-PTEN-3′-UTR-MT lacked the predicted binding site (PTEN-3′-UTR-MT) (Figure 5A(b)).

Co-transfection of RLuc-PTEN-3′-UTR-WT and miR-222-3p mimics was conducted in both BHP5-16 and K1 cells. Co-transfection with RLuc-PTEN-3′-UTR-WT and miR-nc served as a control. The results of dualluciferase assay system demonstrated that the activity was reduced by nearly 30% in miR-222-3p co-transfection group compared with the control. To provide further evidence that the decrease of luciferase activity in the RLuc-PTEN-3′-UTR-WT vector co-transfected cells was due to direct interaction between the miR-222-3p and its putative binding site, we transfected BHP5-16 and K1 with RLuc-PTEN-3′-UTR-MT (lacking the predicted binding site) (PTEN-3′-UTR-MT) and miR-222-3p mimics to monitor the luciferase activity. The results revealed that the suppression of luciferase activity was completely eliminated in RLuc-PTEN-3′-UTR-MT and miR-222-3p mimics co-transfection cells, proving that the mutation of the target sites of PTEN 3′-UTR is able to block the function of miR-222-3p (Figure 5B). These results suggested that miR-222-3p, as predicted, binds directly to putative PTEN 3′-UTR regions. Furthermore, quantitative RT-PCR (qRT-PCR) and western blot analysis were also applied to monitor the expression level of PTEN in the cell lines transfected with miR-222-3p mimics or inhibitor. QRT-PCR showed that no significant difference of PTEN mRNA level was found in the miR-222-3p mimics transfected cells as compared with cells transfected with miR-nc (Figure 5C). By western blot analysis, we found that up-regulation of miR-222-3p significantly decreased the expression of PTEN while down-regulation of miR-222-3p significantly increased the expression of PTEN (Figure 5D). Taken together, our results confirm that miR-222-3p directly targets PTEN and represses its expression at post-transcriptional level in PTC cell lines, confirming the previous reports [25, 26].

Considering that Gas5 is a target of miR-222-3p, we deduce that Gas5 may serve as a ‘sponges’ or ‘decoys’ for miR-222-3p, modulating the expression of PTEN. Thus, the effect of Gas5 on the expression of PTEN protein was investigated. Western blotting analysis indicated that forced expression of Gas5 in BHP5-16 and K1 cells exerted a promoting role on endogenous PTEN protein expression (Figure 5E). In addition, we further observed the level of PTEN in cells co-transfected with Gas5 and miR-222-3p. The results showed that the PTEN level of the group co-transfected with Gas5 and miR-222-3p was much higher than that of the group transfected with miR-222-3p, while slightly lower than that of the Gas5 transfecting group with no statistical significance (Figure 5F). In conclusion, these data confirm that Gas5 can up-regulate the expression of PTEN in PTC cancer partly by mediate miR-222-3p and control its targets PTEN.

In order to demonstrate that Gas5 modulates the expression of PTEN partly by competitive binding to miR-222-3p in PTC cells, we carried out a luciferase assay. The plasmid pcDNA3.1/Gas5 or pcDNA3.1 was co-transfected with miR-222-3p mimics and RLuc-PTEN-WT 3′UTR into BHP5 -16 and K1 cells. Results showed that in the presence of Gas5, RLuc-PTEN-WT 3′-UTR repression was removed compared with the control group (Figure 5G). These findings indicate that aberrant expression of Gas5, a ceRNA of miR-222-3p, may be involved in the progression of PTC by abolishing the miR-222-3p induced repressing activity on PTEN.

### Gas5 activates PTEN/AKT pathway repressing the proliferation of PTC cells

Next, we further investigated the mechanism of Gas5 in the regulation of the viability of PTC cells. The PI3K/AKT signaling pathway exerts the effect on cell proliferation through its downstream effector AKT. PTEN is a key suppressor of oncogenic PI3K/AKT signaling. Considering the changes of expression level of PTEN, we detected the activity of the PI3K/AKT pathway in cells transfected with Gas5, miR-222-3p mimics, miR-222-3p inhibitor or Gas5 and miR-222-3p mimics respectively. Cells transfected with pcDNA3.1 or miR-nc was considered as the control. The western blot results showed the phosphorylation level of AKT (P-AKT). Just like knocking down miR-222-3p by miR-222-3p inhibitor, forced expression of Gas5 decreased the level of P-AKT as compared with the control group. In contrast, over-expression of miR-222-3p enhanced P-AKT level, which could be reversed by Gas5. No statistical difference of total AKT level was found between the groups (Figure 5D–5F). These data demonstrated that Gas5 can activates PTEN/AKT pathway repressing the proliferation of PTC cells, which may be partly mediated by sponging miR-222-3p.

## DISCUSSION

It is now increasingly acknowledged that lncRNAs might participate in the biological process of cancer. Therefore, detailed molecule mechanism of lncRNA may lead to new insights into pathogenetic process and novel therapy for this important disease [27]. LncRNA-Gas5 was a non-protein coding gene which was preferentially expressed in growth-arrested cells [28]. A large body of research suggested that Gas5 was dysregulated in multiple cancers, such as prostate cancer and breast cancer [29–31] and confirmed a tumor suppressor role for this molecule. However, little is known regarding its expression and function in thyroid cancer.

In this research, we first conducted a review of previous microarray data in Oncomine database (www.oncomine.com) for the expression of Gas5 on mRNA level in human PTC. And then we examined the expression level of Gas5 in papillary thyroid carcinoma cells and identified the function of Gas5 by applying forced expression approaches. We confirmed that Gas5 was downregulated in papillary thyroid carcinoma clinical samples as well as in PTC cell lines, which was consistent with the recently reported findings of Guo *et al.* [32]. Over-expression of Gas5 significantly inhibited cell proliferation ability *in vitro* and *in vivo* manifested by MTT, colony formation assays and tumor formation assays. Thus, we may deduce that Gas5 may represent a potential biomarker and a new therapeutic target for PTC, which prompts us to explore the detailed mechanisms by which Gas5 inhibits thyroid carcinogenesis and PTC progression, stimulates novel research directions in PTC.

Sitimulated by the ceRNA regulatory circuitry and more and more data that lncRNAs may be involved in this regulatory network, we hypothesized that Gas5 may also act as a ceRNA exerting its biological function in PTC. To explore the correlation between miRNA and Gas5 in PTC pathogenesis, we employed bioinformatics analysis and found that the miR-222-3p had a higher score binding to Gas5, there followed a further study with a particular focus on the miR-222-3p’s target gene PTEN. Luciferase assays of this case verified a novel ceRNA regulatory network, Gas5/miR-222-3p /PTEN. Consistent with Gas5 sequestration of miR-222-3p, we found that Gas5 over-expression restored PTEN protein synthesis in cells treated with miR-222-3p.

PTEN is a key suppressor gene of tumor cell growth by inhibiting the phosphorylation of AKT. In this study, dual-luciferase assay and western blot verified that PTEN was one target of miR-222-3p. Forced expression of miR-222-3p by mimics transfecting significantly decreased the level of PTEN, accompanied by an remarkably increasing level of p-AKT while miR-222-3p knockdown by inhibitors showed an obviously opposite results. Previous researches reported that miR-222 played multiple roles in cancers acting as an onco-miR [33–35] or a tumor suppressor-miR [36, 37] depending on their target genes. In PTC, Pallante [38] reported that expression of miR-221,-222 and-181b had 5-to 35- fold differential in FNAB samples of PTCs compared with other thyroid nodules. Furthermore, together with other miRNAs, miR-222 is considered as a signature that differentiates malignant from benign indeterminate thyroid lesions [39], the degree of aggressiveness of papillary thyroid carcinoma [40] or a circulating biomarker of recurrent papillary thyroid cancer [41]. In our study, we confirmed that miR-222-3p can promote the proliferation of PTC cells by targeting PTEN consequently activating PTEN/AKT pathway. At the same time, over-expression of Gas5 in PTC cells lead to a contrary result, that is, a higher level PTEN and a lower level p-AKT as compared with control. Furthermore, Gas5 can reverse the effects of miR-222-3p. Therefore, we may reasonably propose that Gas5, acting as a ceRNA , exerts a miRNA/lncRNA trans-regulatory effect on protein-coding mRNAs. Therefore, Gas5 may be a potential prognostic marker and therapeutic target.

However, it should be considered that the ceRNA activity of Gas5 may enable it to sponge a mass of miRNAs, while one miRNA is likely to regulate multiple genes as well. Therefore, the cellular phenotypes we observed are likely due to simultaneous targeting of multiple targets in PTC. Besides, there may be many other lncRNAs functioning as ceRNAs involved in the pathogenesis of PTC. Thus, the identification of these ceRNAs will ultimately promote the progression of lncRNA-directed diagnostics and treatment against PTC.

**Figure 6:**
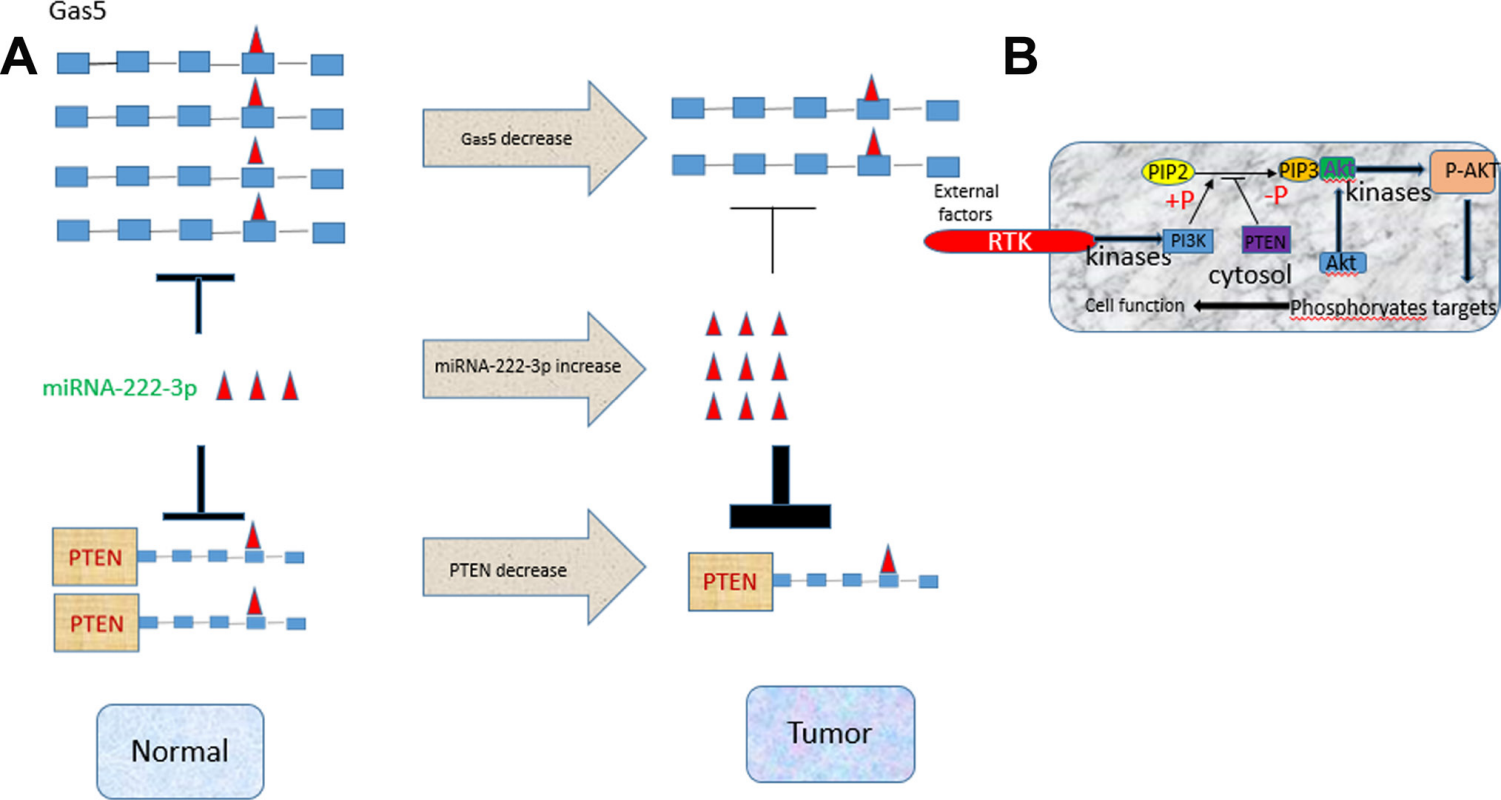
An illustrative figure detailing the relationship among Gas5, miR-222-3p and PTEN (**A**) High expression of both Gas5 and PTEN, low expression of miR-222-3p in normal cells. In tumor cells low expression of Gas sponges less miR-222-3p that is highly expressed leading to a stronger inhibition of miR-222-3p on PTEN. (**B**) A detailed description on the PTEN/AKT-PTEN signaling pathway. Low expression of PTEN dephosphorylates less PIP3, resulting in an enhanced level of phosphorylation of AKT.

Taken together, our study provides experimental evidence that Gas5 functions as a competing endogenous RNA to regulate PTEN expression by sponging miR-222-3p in papillary thyroid carcinoma (Figure 6). This finding may offer a new potential targeting therapy for the treatment of PTC.

## MATERIALS AND METHODS

### Oncomine database analysis

The informatics data on Gas5 mRNA expression in papillary thyroid carcinoma were obtained from the Oncominedatabase (https://www.oncomine.org, Rhodes *et al.*, 2004). He *et al.* (GEO accession GSE3467) datasets were used to compare the expression levels of Gas5 between PTC and normal thyroid tissues.

### Cell culture and reagents

Four papillary thyroid carcinoma cell lines (BHP5-16, TPC, K1 and BHP2-7) and a normal human thyroid epithelium cell line (Nthy-ori 3-1) were used in this study. Among them, BHP5-16 and BHP2-7 were donated by Pro. Tenglisong from Zhejiang university, China. K1, TPC and Nthy-ori 3-1 were donated by Pro. Yuyang from cancer hospital, Tianjin medical university, China. Cells were cultured in RPMI 1640 (Invitrogen) supplemented with 10% fetal bovine serum(Gibico) 100 U/ml penicillin, and 100 mg/ml streptomycin(Invitrogen) in humidified air at 37°C with 5% CO_2_. Hsa-miR-222-3p mimics, hsa-miR-222-3p inhibitor, pcDNA3.1/Gas5 plasmid and negative control miRNA (miR-nc) were chemically synthesized by Shanghai Integrated Biotech Solutions Co., Ltd. (Shanghai, China).

### Transient transfection

Cells were seeded in 6-well dishes the night before to give 80∼90% confluence for plasmid and 40∼50% for miRNA at the day of transfection. The following day cells were transfected with miR-222-3p mimics (50 nM), miR-222-3p inhibitor (100 nM), miR-nc (50 nM), inhibitor-nc (100 nM), pcDNA3.1/Gas5 (4.0μg) or pcDNA3.1 (4.0 μg) respectively using Lipofectamine 2000 (invitrogen), according to the manufacturer’s instructions. After transfection for 6 hours, the medium was replaced with normal culture medium.

### RNA extraction and qRT-PCR analyses

Total RNA extraction was conducted using a TRIzol reagent kit (Life Technologies, Inc., Rockville, MD, USA) according to the manufacture. Then, total RNA was reversely transcribed to complementary DNA (cDNA) using a reverse transcription kit (Thermo). QRT-PCR reactions were performed with EvaGreen 2 × qPCR (abm) using LightCycler^®^ 96 (Roche). The relative expression of Gas5 mRNA and PTEN mRNA was calculated using the 2-ΔCT method normalized to GAPDH. The reaction conditions of PCR were as follows: 95°C pre-denaturation for 2 min followed by 40 cycles of 95°C denaturation for 15 s, 60°C annealing for 30 s, and 72°C extension for 30 s. For miR-222-3p expression detection, Stem-loop qRT-PCR Detection Kit (GenePharma) was used and U6 snoRNA was validated as the normalizer. Primer sequences were as follows: Gas5 forward 5′-AGCTGGAAGTTGAAATGG-3′and reverse 5′-CAAGCCGACTCTCCATA C-3′, PTEN forward 5′-TGGAAAGGGACGAACTGGTG-3′, reverse5′-CATAGCGCCTCTGA CTGGGA-3′, GAPDH forward 5′-ACCCACTCCTCCACCTTT-3′, reverse 5′-GCTGTAGCCAAATTCGTTGT-3′.

### Cell proliferation assay

Transfected PTC cells were seeded into 96-well plates in a density of 5 × 10^3^ cells per well. At 24, 48, 72 and 96 h, culture medium was replaced with fresh medium containing MTT dye (20 μl per well, Solarbio, Beijing) was added to each well and incubated 4 h at 37°C. After removing the medium, dimethyl sulfoxide (DMSO) (150 μl per well; Sigma, USA) was added and mixed for 10 min. The absorbance was detected by Universal Microplate Spectrophotometer (Bio-Tek Instruments, Inc., Winooski, VT, USA). OD570nm value was measured.

### Colony formation assay

Seventy-two hours after transfection, cells were trypsinized (Solarbio, Beijing), replaced in 6-well plates at 200 cells per well and cultured in RPMI 1640 (Invitrogen) supplemented with 10% fetal bovine serum (Gibico) under routine conditions for 21 days. Medium was changed if needed. Then colonies were fixed with methanol and stained with 0.5% crystal violet (Sigma). Colonies were counted. Visible colonies were manually counted.

### Tumor formation assay

Female BALB/c nude mice were purchased at 5–6 weeks of age from Beijing Vital River Laboratory Animal Technology Co., Ltd. For xenograft models, 5 × 10^5^ BHP5-16 cells transfected with pcDNA3.1/Gas5 or pcDNA3.1 were subcutaneously injected in the bottom right back of BALB/c nude mice respectively. Tumor growth was examined every 3 days when the implantations were starting to grow bigger. After 6 weeks the mice were sacrificed, necropsies were performed and the tumor tissues were harvested. Tumor volumes were calculated as described previously.

### Dual-luciferase assay

Cells (2.0 × 10^4^) grown in a 96-well plate were co-transfected with 150 ng of either MiR-nc or miR-222-3p mimics, 50 ng of psi-check Luciferase Expression Reporter (Promega) comprising 3′UTR of Gas5 (wild type or mutant type) for testing the relationship between miR-222-3p mimics and Gas5 plasmid. Cells co-tansfected with 150 ng of miR-222-3p mimics, 50 ng of pEZX-MT01 Luciferase miRNA Expression Reporter (GeneCopeia) comprising 3′UTR of PTEN (wild type or mutant type) were used for evaluation the relationship between miR-222-3p and PTEN. In addition, cells co-tansfected with 150 ng of miR-222-3p mimics, 25 ng of pEZX-MT01 Luciferase miRNA Expression Reporter (GeneCopeia) comprising 3′UTR of PTEN (wild type), 75 ng of Gas5 plasmid or empty vector were used to determine the relationship among miR-222-3p, PTEN and Gas5. Cells were harvested 48 h after transfection for luciferase assay using a luciferase assay kit (Promega) on a Synergy 2 Microplate Reader Fluorometer (BioTek) according to the manufacturer’s instruction. The relative luciferase activity was calculated as the ratio of firefly luciferase activity versus renilla luciferase activity.

### Western blot

Western blot analysis were conducted following standard protocols as described earlier [42]. Briefly, whole cell lysates were prepared and protein concentrations were quantified colourimetrically using a BCA Protein Assay Kit (Solarbio, Beijing). Samples were separated in a 10% SDS-polyacrylamide gel (Solarbio, Beijing) and blotted onto a PVDF membrane (Millipore). Immunoblot was performed with commercially available antibodies (anti PTEN antibody, 1:5000, Santa Cruz; anti- AKT antibody, 1:1000, BD Bioscience; anti-P-AKT antibody, 1:1000, Cell Signaling Technology; anti-GAPDH antibody, 1:5000, Santa Cruz.). ECL (Millipore) was applied for chemiluminescence detection. Immunoblot signal quantifications were performed using Image J software.

### Statistical analysis

All data were expressed as mean ± standard from three independent experiments. The independent experiments need to be at least three times. Statistical analysis was performed using ANOVA with SPSS 19.0 software (IBM Corporation, Armonk, NY, USA). The *P* < 0.05 were considered statistically significant.
